# Prior Expectations of Motion Direction Modulate Early Sensory Processing

**DOI:** 10.1523/JNEUROSCI.0537-20.2020

**Published:** 2020-08-12

**Authors:** Fraser Aitken, Georgia Turner, Peter Kok

**Affiliations:** Wellcome Centre for Human Neuroimaging, UCL Queen Square Institute of Neurology, University College London, London WC1N 3AR, United Kingdom

**Keywords:** magnetoencephalography, perceptual inference, prediction, sensory processing, top down modulation, visual perception

## Abstract

Perception is a process of inference, integrating sensory inputs with prior expectations. However, little is known regarding the temporal dynamics of this integration. It has been proposed that expectation plays a role early in the perceptual process, biasing sensory processing. Alternatively, others suggest that expectations are integrated only at later, postperceptual decision-making stages. The current study aimed to dissociate between these hypotheses. We exposed human participants (male and female) to auditory cues predicting the likely direction of upcoming moving dot patterns, while recording neural activity using magnetoencephalography (MEG). Participants' reports of the moving dot directions were biased toward the direction predicted by the cues. To investigate when expectations affected sensory representations, we used inverted encoding models to decode the direction represented in early sensory signals. Strikingly, the cues modulated the direction represented in the MEG signal as early as 150 ms after visual stimulus onset. While this may not reflect a modulation of the initial feedforward sweep, it does reveal a modulation of early sensory representations. Exploratory analyses showed that the neural modulation was related to perceptual expectation effects: participants with a stronger perceptual bias toward the predicted direction also revealed a stronger reflection of the predicted direction in the MEG signal. For participants with this perceptual bias, a correlation between decoded and perceived direction already emerged before visual stimulus onset, suggesting that the prestimulus state of the visual cortex influences sensory processing. Together, these results suggest that expectations play an integral role in the neural computations underlying perception.

**SIGNIFICANCE STATEMENT** Perception can be thought of as an inferential process in which our brains integrate sensory inputs with prior expectations to make sense of the world. This study investigated whether this integration occurs early or late in the process of perception. We exposed human participants to auditory cues that predicted the likely direction of visual moving dots, while recording neural activity with millisecond resolution using magnetoencephalography. Participants' perceptual reports of the direction of the moving dots were biased toward the predicted direction. Additionally, the predicted direction modulated the neural representation of the moving dots just 150 ms after they appeared. This suggests that prior expectations affected sensory processing at early stages, playing an integral role in the perceptual process.

## Introduction

Since Helmholtz (1867) described perception as a process of 'unconscious inference', it has become widespread to conceptualise perception as an integration of bottom-up sensory information with top-down prior expectations (Kersten and Yuille, 2003; Friston, 2005; Summerfield and de Lange, 2014). However, the neural mechanisms and time course of this integration remain controversial.

On the one hand, influential theories of predictive processing, such as predictive coding (Rao and Ballard, 1999; Friston, 2005), posit that top-down predictions are integrated with bottom-up sensory information from the moment inputs arrive in thecortex, such that sensory representations are modulated by expectations at early sensory stages (Rao and Ballard, 1999; Lee and Mumford, 2003; Friston, 2005; Wyart et al., 2012; Keller and Mrsic-Flogel, 2018). In support of this hypothesis, many studies have shown that prior expectations can modulate processing at the earliest stages of the cortical hierarchy (Den Ouden et al., 2009; Alink et al., 2010; Kok et al., 2012), as well as early in time (starting around 100–150 ms post-stimulus; Todorovic et al., 2011; Wacongne et al., 2011; Hsu et al., 2015; Aru et al., 2016; Jabar et al., 2017; Alilović et al., 2019 or even as early as 75 ms; Keil et al., 2017), even prior to stimulus presentation (Sherman et al., 2016; Kok et al., 2017).

Alternatively, it has been suggested that prior expectations leave early sensory processing untouched, and instead only modulate later decision-making processes (Rao et al.,2012; Bang and Rahnev, 2017; Rungratsameetaweemana et al., 2018), for instance in parietofrontal brain circuits (Heekeren et al., 2004; Gold and Shadlen, 2007). Under this account, the effects of expectations in early sensory regions as revealed by previous functional magnetic resonance imaging studies are proposed to reflect late, postdecision feedback signals, simply “informing” sensory regions of the decision that has been made. Even early effects of expectations revealed by electrophysiological studies (Chaumon et al., 2008; Gamond et al., 2011; Todorovic et al., 2011) may be epiphenomena rather than directly impacting perception, analogous to the proposals that working memory representations in sensory regions reflect epiphenomena (Xu, 2018; but see Zhang et al., 2019).

Previous studies have been unable to distinguish between these two hypotheses, since they have not linked the neural effects of expectation to behavioral changes in perception. Additionally, most previous electrophysiological studies measured the overall amplitude of the neural response to expected or unexpected stimuli, rather than probing stimulus-specific representations in the neural signal (Aru et al., 2016; Rungratsameetaweemana et al., 2018). This is critical, since previous studies have shown that informational content can be fully dissociated from the overall amplitude of neural signals (Harrison and Tong, 2009; Kok et al., 2012). Therefore, these studies may have missed stimulus-specific effects of expectations on sensory processing. A notable exception is the study by Alilović et al. (2019), who found that spatial expectations affected stimulus location representations starting at ∼200 ms poststimulus.

Here, we overcame these limitations by using magnetoencephalography (MEG) to directly relate the effects of expectation on neural representations to the effects on the contents of perception. Participants were exposed to auditory cues that, unbeknownst to them, predicted the likely motion direction of a subsequent random dot kinetogram (RDK). Perception was probed by asking participants to report which direction the dots were moving in. A forward model decoder (Brouwer and Heeger, 2009; Kok et al., 2017), trained on task-irrelevant RDKs presented in independent runs, was used to reveal the motion direction represented in the MEG signal immediately after stimulus presentation. This allowed us to determine the time points at which the sensory representation was modulated by the prediction cue.

To preview, we found that prior expectations modulated the content of sensory representations as early as 150 ms poststimulus. These neural effects were mirrored by a bias in perception, in line with proposals that expectations can bias perception by modulating early sensory processing.

## Materials and Methods

### 

#### 

##### Participants.

Thirty healthy human volunteers participated in the MEG experiment. The study was approved by the UCL Research Ethics Committee, and all participants gave informed consent and received monetary compensation. Two participants were excluded because of excessive head movement, one for excessive eye blink artifacts during stimulus presentation, one because >50% of trials had to be rejected because of artifacts, and two because of below-threshold task performance (*r* < 0.9 between mean perceived and presented direction). The remaining 24 participants (11 female; mean ± SD age, 25 ± 8) had normal or corrected-to-normal vision. This sample size was chosen on the basis of similar previous studies that had observed significant effects (Kok et al., 2013, 2017; Mostert et al., 2015).

##### Stimuli.

All stimuli were generated using MATLAB (MathWorks; RRID:SCR_001622) and the Psychophysics Toolbox (David Brainard, Department of Psychology University of California, Santa Barbara, Santa Barbara, CA; RRID:SCR_002881). The visual stimuli were RDKs, which consisted of white dots (dot size, 0.1° visual angle; density, 2.5 dots/°2) on a gray background. Each RDK display contained a given proportion of dots moving in a coherent direction, with the remaining dots moving in random directions. Each dot appeared at a random location, moved at a speed of 6°/s, and lasted for 200 ms before disappearing. The dots were displayed in an annulus (inner diameter, 3°; outer diameter, 15°), surrounding a white fixation bullseye (diameter, 0.7°) for 1 s. The auditory stimuli consisted of pure tones (450 or 1000 Hz) and lasted 200 ms.

During the behavioral session, visual stimuli were presented on an LCD monitor (1024 × 768 resolution; 60 Hz refresh rate), and tones were presented on external speakers. During the MEG session, visual stimuli were projected on a screen placed 58 cm from the participants' eyes (1024 × 768 resolution; 60 Hz refresh rate), and auditory stimuli were presented via earphones inserted into the ear canal (E-A-RTONE 3A 10 Ω, Etymotic Research).

##### Experimental procedure.

**Figure 1.**
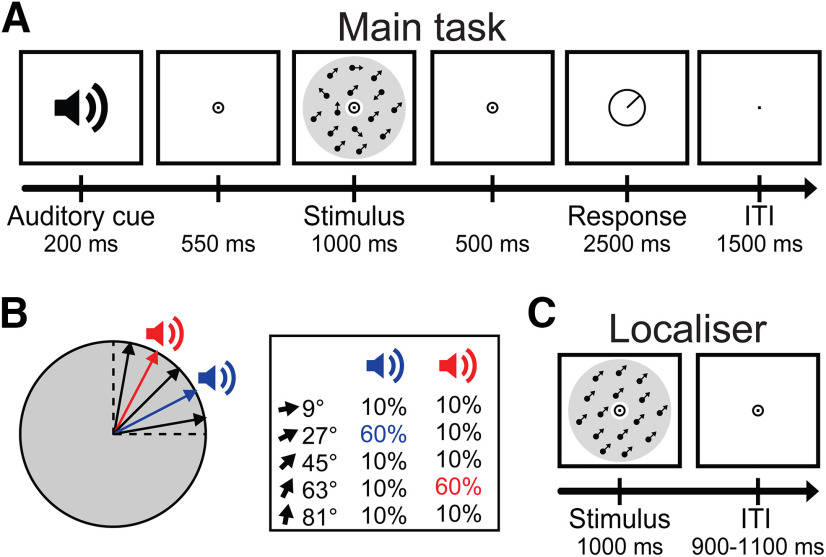
Schematic diagram of the experimental procedure. ***A***, The main task. Participants were presented with an auditory cue, followed by an RDK stimulus. Participants indicated their response on a continuous scale by rotating a line segment. ***B***, Possible coherent directions in the RDK. One tone predicted 27°, and the other tone predicted 63°, each with 60% validity. ***C***, In localizer runs, participants were presented with task-irrelevant moving dots stimuli with 100% coherence, while performing a dot-dimming task at fixation.

The experiment consisted of two types of task runs. In the main task, each trial consisted of an auditory cue (200 ms) followed after 550 ms by a visual RDK stimulus for 1000 ms (Fig. 1*A*). After a 500 ms interval, participants reported the direction of the coherently moving dots by orienting a line segment in a 360° circle (2500 ms). The initial direction of the line was randomized between −45° and 135°. After the response interval, during the intertrial interval (ITI; 1500 ms), the fixation bullseye was replaced by a single dot, signaling the end of the trial while still requiring participants to fixate. The RDKs had one of the following five possible directions of coherent motion: 9°, 27°, 45°, 63°, or 81°. Participants were informed that the coherent direction would range between 0° and 90°, but not that there was a discrete set of possible directions. The two auditory cues predicted either 27° or 63°, respectively, with 60% probability (Fig. 1*B*). Participants were not informed of this cue–direction relationship. The four nonpredicted directions were each equally likely to occur (10% probability). The relationship between which tone predicted which direction was counterbalanced across participants. Thus, for example, for half the participants, a 1000 Hz auditory cue would indicate that 27° would be presented with 60% probability, and that 9°, 45°, 63°, and 81° would each be presented with 10% probability, while a 450 Hz auditory cue would predict 63° with 60% probability, and 9°, 27°, 45°, and 81° each with 10% probability. For the other half of the group, the cue–direction contingencies were opposite, meaning a 450 Hz cue predicted 27° and a 1000 Hz cue predicted 63°. Note that, as a result of this, 27° and 63° motion directions were presented more often over the course of the experiment than 9°, 45°, and 81°. Participants were not informed of the cue–direction relationships, or of the fact that 27° and 63° occurred more often than other directions overall. A debrief questionnaire at the end of the experiment asked participants whether they had consciously noted either of these aspects of the experiment (see below for details). Each run contained 60 trials (∼6 min).

During localizer runs, RDKs were presented with 100% coherence, creating training data for the MEG decoder (Fig. 1*C*). Eleven motion directions were presented in a pseudorandom order, for 1000 ms each. These directions were –45°, −27°, −9°, 9°, 27°, 45°, 63°, 81°, 99°, 117°, and 135°. One localizer block consisted of 88 trials (∼3 min). The fixation bullseye at the center of the annulus dimmed at random time points, and subjects were instructed to press a button when this occurred. The ITI was jittered between 900 and 1100 ms. During these runs, the moving dots were fully task irrelevant in order to extract motion direction signals independent of task demands (Kok et al., 2017). The task was also intended to encourage central fixation in order to minimize eye movement-related confounds (Mostert et al., 2018).

All participants took part in a behavioral session 1–4 d before the MEG session to familiarize them with the task and expose them to the cue–direction contingencies. Participants received written instructions and performed two short blocks (of 20 and 40 trials, respectively) with trial-by-trial feedback to facilitate learning. They then performed seven main task blocks of 60 trials each (∼45 min) during which they no longer received trial-by-trial feedback, but were informed of their mean error after each block for motivation, as in the MEG session. The RDKs began with 40% coherence in the instructions and practice blocks, to facilitate learning of the task, and gradually reduced from 40% to 20% coherence during the main behavioral session. Finally, participants participated in one localizer block to familiarize them with the fixation dimming task. In the MEG session, participants performed five to seven runs (∼9 min each). Each run consisted of 60 trials of the main task, followed by a 15 s pause, then one block of the localizer task. In the main task, the RDKs had 20% coherence. After the experiment, participants filled out a debriefing questionnaire to verify the implicit nature of the expectations. They were asked: “Did any directions of motion occur more often than the rest? If so, please indicate which direction(s) you thought occurred more often than the others.” Subsequently, participants were asked, “Did you notice any relationship between the tones you heard and the directions of motion you saw? If so, please describe the relationship you observed in the text box below.” For both questions, they were also provided with a unit circle in which they could illustrate their answer by drawing arrows to represent specific motion directions.

Most participants (22 of the 24) reported noticing that some directions occurred more often than others. An inspection of their drawings indicates that of these participants, seven correctly reported that 27° and 63° occurred most often. More importantly, a minority (7 of 24 participants) reported noticing a relationship between the tones and directions. Of these, four participants reported the correct relationship, two reported the opposite relationship, and one did not report any specific relationship. We replicated our main MEG analyses with the four participants who reported the correct relationship excluded (see Results).

##### MEG recording and preprocessing.

Whole-head magnetic signals were recorded continuously (600 Hz sampling rate) using a MEG system (CTF) with 272 functioning axial gradiometers inside a magnetically shielded room. Participants were seated upright and indicated their responses on an MEG-compatible button box. To minimize eye blink-related artifacts, participants were instructed to blink only when the RDK was not on the screen. Eye movement was recorded using an EyeLink 1000 eye tracker (1000 Hz sampling rate). Presentation latencies for stimuli (visual, ∼17 ms; auditory, ∼15 ms) were measured using a photodiode and microphone; these were used to align the MEG and eye-tracking data to the onset of stimulus presentation. After the first MEG run, participants were informed of their head motion and encouraged to stay as still as possible during the recordings. Since participants displayed substantially more head motion during the first run, this run was discarded for all participants.

The data were preprocessed using FieldTrip (Oostenveld et al., 2011). To detect irregular artifacts, the variance, collapsed over channels and time, was calculated for each trial. Trials with large variances were visually inspected and removed if they contained large and irregular artifacts. Trials with eye blinks during RDK presentation were also removed. This resulted in the removal of 71 ± 47 (mean ± SD; ∼14 ± 9%) trials from the localizer runs, and 26 ± 20 (∼7 ± 5%) trials from the main task runs. Independent component analysis (ICA), using the logistic infomax ICA algorithm as implemented in the EEGLAB toolbox (https://sccn.ucsd.edu/eeglab/), was used to remove regular artifacts, by correlating the independent components (ICs) with the eye-tracking data to identify eye blinks, and then manually inspecting before removing ICs related to eye blinks. Twenty-two of 24 participants had one IC removed from each MEG run, 1 participant had two ICs removed from one run and one IC from the remaining runs, and 1 participant had three ICs removed from one run, 2 ICs from another run, and 1 IC from the remaining runs. Data were low-pass filtered with a two-pass Butterworth filter with a filter order of 6 and a cut off of 40 Hz. The data were baseline corrected on the interval of −250 to 0 ms relative to auditory cue onset for the main task, and −200 to 0 ms relative to visual onset for the localizer task.

##### Decoding analysis.

To probe the effect of expectations on stimulus representations in visual cortex, we used a forward modeling approach (Brouwer and Heeger, 2009) to decode motion directions from the MEG signal (Myers et al., 2015; Kok et al., 2017). This approach has been highly successful at decoding continuous stimulus features from neural data (Brouwer and Heeger, 2009, 2011; Garcia et al., 2013; Kok et al., 2013, 2017; Myers et al., 2015). Furthermore, it yields decoded features on a continuous dimension rather than a discrete classification, making it potentially more sensitive to subtle biases than a categorical classifier.

The decoding approach consisted of two stages. First, the model was trained on the MEG data from the moving dots localizer to create an encoding model: a transformation from stimulus (motion direction) space to MEG sensor space. Then, this encoding model was inverted to create a decoding model, which was used to transform unseen MEG data (from the main task runs) from sensor space to motion direction space. Thus, the decoding model was estimated on the basis of the moving dot localizer data, and then applied to the data from the main experiment to generalize from sensory signals evoked by task-irrelevant moving dots to the noisy moving dot signals evoked in the main task (Kok et al., 2017). To test the performance of the model, we also applied it to the localizer data using the following cross-validation approach: in each iteration, one run of the localizer was used as the test set, and the remaining data were used as the training set.

The forward encoding model consisted of 21 hypothetical channels, each with an idealized direction tuning curve: a half-wave rectified sinusoid raised to the sixth power. The 21 channels were spaced evenly within the 180° space ranging from −45° to 135° to cover all directions presented in the localizer runs. For each participant, the MEG data from the localizer were used to calculate an encoding model. First, a matrix $C_{train}$ (21 channels × $n_{train}$ trials) was generated, containing the hypothesized channel amplitudes for each trial. Specifically, each row of matrix $C_{train}$ was calculated by expressing the presented direction as a hypothetical amplitude for each channel, resulting in the row vector $c_{train,i}$ of length $n_{train}$ for each channel *i*. The sensor data were represented in a matrix $B_{train}$ (272 sensors × $n_{train}$ trials). The key aspect of the encoding model was a weight matrix, specifying the transformation from stimulus space (represented in matrix $C_{train}$) to sensor space (matrix $B_{train}$). The rows of the weight matrix were calculated by least-squares estimation for each channel, as follows:

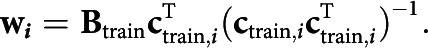


This was used to create the following linear encoding model:

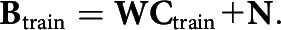


Here, W is a weight matrix (272 sensors × 21 channels) specifying the transformation from stimulus representational space (channel activities) to neural representational space (sensor amplitudes). N represents the residuals.

In the second stage of the analysis, the decoding model was created by inverting the encoding model. This was achieved using a recently developed method taking the noise covariance between (neighboring) sensors into account, which increases decoding accuracy compared with a decoding model that does not adjust for noise covariance (Mostert et al., 2015; Kok et al., 2017). First, $B_{train}$ and $C_{train}$ were demeaned, so that their average over trials was 0 for all sensors and channels, respectively (Kok et al., 2017). Then, noise covariance, $\Sigma_{i}$, between the sensors was estimated for each channel i using the following equations:

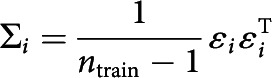


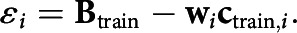


To optimize noise suppression, regularization by shrinkage, using analytically determined optimal shrinkage parameters, was used to calculate regularized covariance matrices for each channel, $\Sigma_{i}^{*}$ (Blankertz et al., 2011, for details). These regularized covariance matrices were used to create spatial filters. The optimal spatial filter $v_{i}$for the *i*th channel was estimated as follows (Mostert et al., 2015; Kok et al., 2017):

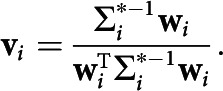


Each filter was normalized so that the magnitude of its output matched the magnitude of the channel activity that it would be used to recover. The filters were combined into a decoding weight matrix V (272 sensors × 21 channels). This decoding weight matrix could then be used to estimate the channel responses for independent MEG data, as follows:

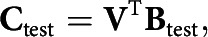
 where $B_{test}$ (272 sensors × $n_{test}$ trials) represents the independent test data.

These channel responses were estimated at each time point of the test data in steps of 5 ms, with the data being averaged within a window of 28.3 ms at each step. The length of 28.3 ms was based on an a priori window of 30 ms, subtracting one sample such that the window contained an odd number of samples and could be centered symmetrically. To verify the ability to decode motion direction from the MEG signal, we first applied the decoding approach to the localizer data themselves in a “leave-one-run-out” cross-validation method. In each iteration, all localizer runs but one were used to estimate the decoding model, which was then applied to estimate the channel responses in the remaining run. The estimated channel responses were used to compute a weighted average of the 21 basis functions, and the direction at which the resulting curve reached its maximum value constituted the decoded motion direction.

Decoding performance was quantified, per time step, by calculating the within-participant Pearson correlation among the 11 presented directions and the mean decoded direction per presented direction. This yielded a correlation coefficient for each participant at every time step. The Pearson correlation was used because we expected to find a linear relationship between variables on an interval scale. However, because of the relatively small sample sizes underlying these correlation coefficients (*N* = 11 directions), it would not have been appropriate to test the significance of these within-participant correlations. Rather, we used these correlations as an index of the linear relationship between the variables and tested the significance of the correlations at the group level. Specifically, we applied Fisher's *r*-to-*Z* transform (Fisher, 1915) to the correlation values, and tested whether they were significantly different from zero at the between-participant level using cluster-based permutation tests. To confirm that these results were reliable, we replicated the analysis using Spearman's ρ, a nonparametric measure of rank correlation, which yielded similar results with a very similar effect size (both analyses revealed a significant cluster from 90 to 110 ms; Spearman's ρ: *p* = 0.0193, *d* = 0.70; Pearson's *r*: *p* = 0.018, *d* = 0.71).

This was repeated with each iteration leaving a different localizer run out, and the final decoding performance was quantified by averaging results across all these iterations. Using cluster-based permutation, we determined the earliest cluster of time points at which decoding performance was significantly above chance at the group level (90–110 ms poststimulus; see Fig. 3). Within this cluster, the peak in decoding performance at the group level was 100 ms. We determined each participant's individual decoding peak within 10 ms of this group peak (i.e., 90–110 ms poststimulus, matching the significant group cluster). For each participant, the final decoding model was trained on this individual peak time point (plus the two neighboring time points on either side, for robustness) in the localizer data, to optimize the detection of early sensory signals. This decoding model was applied to the data from the main task in steps of 5 ms, with the data being averaged within a window of 28.3 ms at each step. As before, the decoded motion direction was calculated as the peak of the curve generated by taking a weighted average of the basis functions, with the estimated channel responses constituting the weights. This procedure yielded a 2D matrix (time × $n_{test}$) specifying the estimated motion direction for each trial in the main experiment, in a time-resolved manner.

##### Statistical analysis.

**Figure 2.**
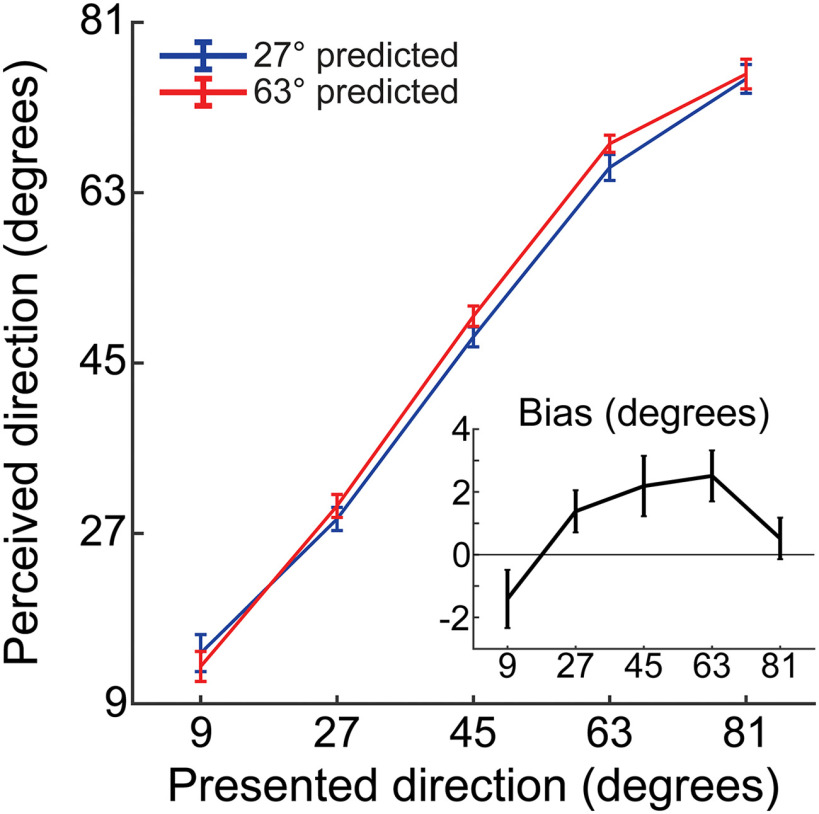
Behavioral results. Reported direction as a function of presented direction. Mean reported direction plotted against presented direction, separately for the two predictive cues. Inset shows the difference in mean reported direction between the two cue conditions. Given that the only difference between these two conditions was the cued direction, this value can be interpreted as the bias induced by the predictive cue for each of the five presented directions separately. Error bars indicate the SEM.

To quantify overall behavioral performance, the mean of all reported directions per presented direction was calculated for each participant (Fig. 2*A*), and was used to calculate the Pearson's correlation coefficient between reported and presented directions, per participant. To ensure the robustness of our results, we also repeated this analysis using Spearman's ρ, which yielded a very similar correlation (mean ± SD: Pearson's correlation = 0.98 ± 0.022; Spearman's ρ = 0.98 ± 0.041). Participants with a coefficient <0.9 (*N* = 2) were excluded from further analysis.

To examine the effects of the predictive cues on reported direction, we performed a two-way repeated-measures ANOVA with factors “Presented Direction” and :Predicted Direction.” There were five presented directions (9°, 27°, 45°, 63°, and 81°) and two predicted directions (27° and 63°), yielding independent variables with five and two levels, respectively. This was to establish the extent to which the direction which the participants reported seeing (Reported Direction; the dependent variable) was affected by what they were actually presented with (Presented Direction), and what the cue predicted (Predicted Direction). Significant main effects and interactions were followed up with *t* tests.

Our main research question was whether prior expectations modulated the neural representation of the moving dot stimuli, and, if so, at which time points. To address this question, we first averaged decoding results over trials, per presented and predicted directions, and then performed a linear subtraction of the decoded direction in conditions where 27° was cued (half of all trials) from the decoded direction in conditions where 63° was cued (the remaining half of trials). The logic behind this method was that, as the only difference between the conditions was the predicted direction, the subtraction would subtract out any signals in common between the cued conditions and isolate any difference related to the cues. We used cluster-based permutation tests (Maris and Oostenveld, 2007) to establish at which time points this subtraction was significantly different from zero. Specifically, univariate *t* statistics were calculated for time points from −250 to 500 ms relative to moving dots onset, in 5 ms steps, and neighboring elements that passed a threshold value corresponding to a *p* value of 0.05 (one tailed) were collected into clusters. Cluster-level test statistics consisted of the sum of *t* values within each cluster, which were compared with a null distribution created by drawing 10,000 random permutations of the observed data. A cluster was considered significant when its *p* value was below 0.05 (i.e., a cluster of its size occurred in <5% of the null distribution clusters).

To investigate whether the motion direction signals we decoded were directly related to subjective perception, we quantified the perceptual “bias” elicited by the cue. For each participant, the bias was the mean reported direction when 27° was predicted subtracted from the mean reported direction when 63° was predicted. This was interpreted as the perceptual bias induced by the predictive cues because the only difference between these two conditions was the direction predicted by the cues. We performed a *post hoc* split of the participants into two subgroups, on the basis of whether they had a mean positive perceptual bias toward the expectation cues (*N* = 17) or not (*N* = 7). To establish whether neural motion direction signals were related to behavioral changes in perception, analyses were performed on these groups. First, to investigate whether there was a neural–perceptual relationship across participants, we performed a between-participants cluster-based permutation test to see whether neural expectation effects differed significantly between the two groups, which were split on the basis of behavior.

Second, to investigate whether there was a neural–perceptual relationship within participants, we calculated the trial-by-trial partial Pearson correlation between the decoded direction from the MEG data and the perceived direction in each trial, controlling for the presented direction. On average, this correlation was calculated over *N* = 263 trials (SD, ±23 trials) per participant. A separate correlation coefficient was obtained for every decoding time-step within the period from −250 to 500 ms, per participant, resulting in *N* = 24 correlation coefficients for each time step. Statistical tests of correlation coefficients were preceded by applying Fisher's *r*-to-*Z* transform (Fisher, 1915). The resulting time courses of *z* values were subjected to cluster-based permutation tests (using the same parameters as described above) at the group level. These correlations were calculated separately for participants with a positive perceptual bias induced by the expectation cues (*N* = 17) and those without such a bias (*N* = 7), since the former had demonstrably used the expectation cue to inform perception, whereas the latter did not.

## Results

### Behavioral results

To ensure that participants were paying attention to and perceiving the directions of the RDKs, only participants with good performance (correlation between mean reported and presented directions, >0.9; for details, see Materials and Methods) were included in the analysis (final sample: mean ± SD, *r* = 0.98 ± 0.02). In the localizer task, participants correctly detected dimming of the fixation dot with high accuracy (mean ± SD: 95.4% ± 8.9%).

Participants' perceptual reports of the direction of the moving dots was significantly biased toward the directions predicted by the auditory cues (*F*_(1,92)_ = 8.5, *p* = 0.0078, η_p_^2^ = 0.27; Fig. 2). That is, on average, motion direction was perceived as being slightly more vertical when the cue predicted 63° (mean ± SEM: 47.3° ± 0.9°) than when the cue predicted 27° (46.3° ± 1.0°). This indicates that, for identical visual stimuli, perception was partially determined by the predictive auditory cues. The cue-induced bias depended on the direction of the presented moving dots (*F*_(4,92)_ = 3.6, *p* = 0.0084, η_p_^2^ = 0.14), being weakest for close to horizontal (9°) and vertical (81°) directions, and stronger for directions closer to oblique (27° to 63°; Fig. 2).

### Expectations modulate sensory representations as early as 150 ms poststimulus

**Figure 3.**
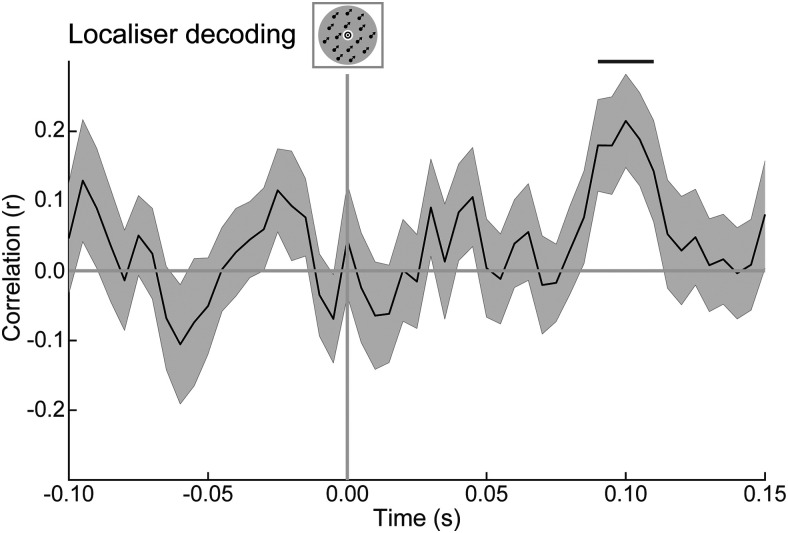
Within-localizer MEG decoding performance. Correlation between presented and decoded direction (cross-validated for each participant, then averaged across participants), plotted for each time point. The shaded region indicates the SEM; horizontal lines represent significant clusters (*p* < 0.05).

**Figure 4.**
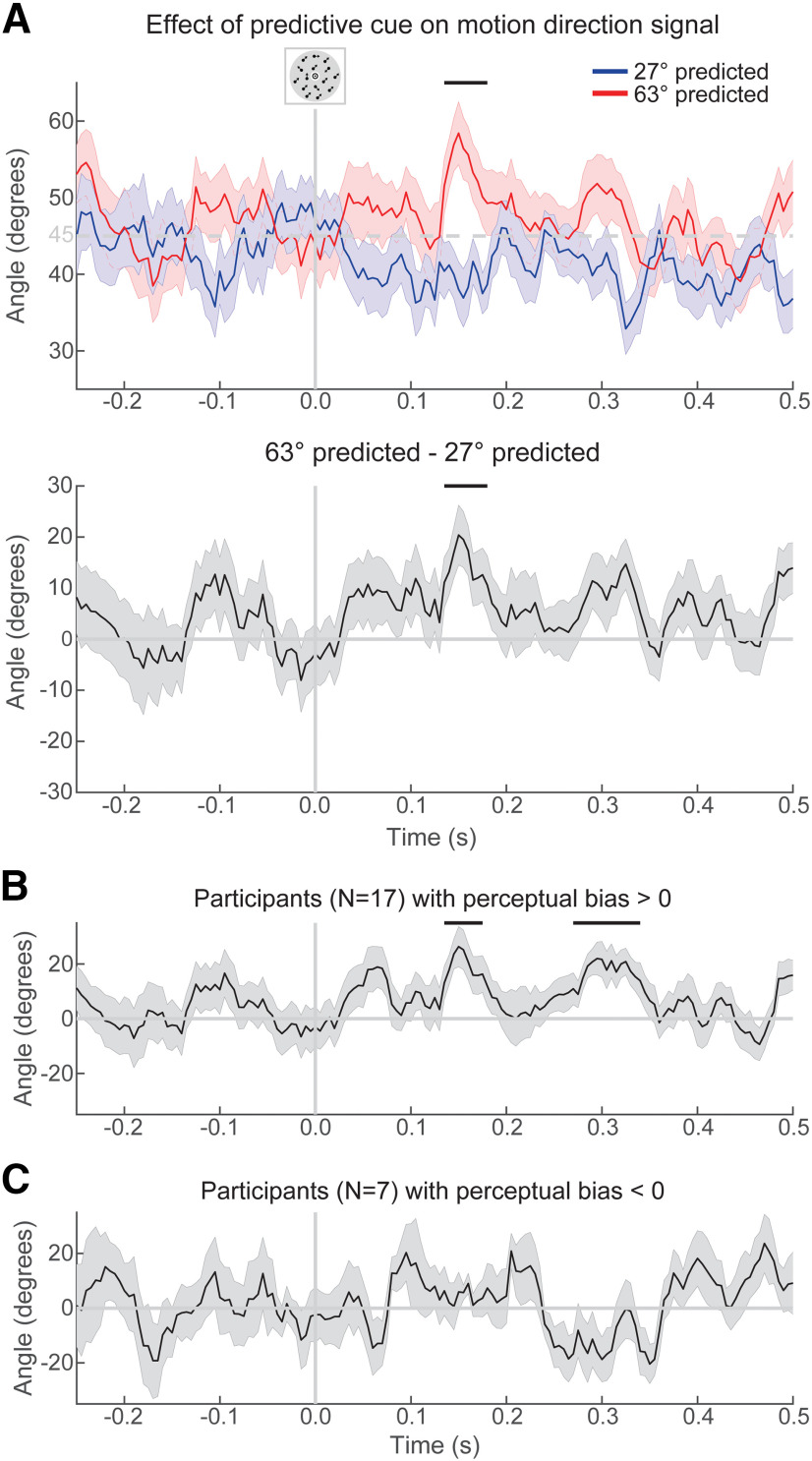
Expectation effects on decoded direction signals. ***A***, Top, The mean decoded direction separately for the trials in which 27° was predicted, and the trials in which 63° was predicted, in blue and red, respectively. Bottom, The cue effects on decoding, obtained as the linear subtraction of the decoded direction between these two cue conditions (i.e., 63° predicted; 27° predicted). ***B***, Cue effects on decoding restricted to participants who showed a perceptual bias toward the predicted directions. ***C***, Cue effects on decoding restricted to participants who did not show a perceptual bias toward the predicted directions. Shaded regions represent the SEM; horizontal lines represent significant clusters (*p* < 0.05).

The first time point at which motion direction could be decoded from the MEG signal evoked by task-irrelevant moving dot stimuli, in the localizer runs, was from 90 to 110 ms poststimulus, peaking at 100 ms (*p* = 0.018, *d* = 0.71; Fig. 3). To probe modulations of early sensory signals by the predictive cues, motion direction-decoding models were trained on participants' individual peaks in this interval in the localizer runs and were used to decode the motion direction from the MEG data in the main task (for details, see Materials and Methods). After obtaining a decoded direction for all trials, the decoded direction in trials where 27° was predicted was subtracted from the decoded direction in trials where 63° was predicted. Therefore, any decoded motion direction signal resulting from this subtraction can only be explained by the difference in predicted directions. This analysis revealed that across the whole group, the predictive auditory cues evoked a significant motion direction signal from 135 to 180 ms poststimulus, peaking at 150 ms (peak difference = 20.4°; *p* = 0.027, *d* = 0.72; Fig. 4*A*).

### Neural expectation signal related to perceptual bias

To relate this neural effect of the predictive cues to perception, we performed an exploratory analysis in which we split the participants into two subgroups on the basis of the direction of their perceptual bias and calculated effects separately for participants who showed a positive perceptual bias (bias = 2.0° ± 1.1°; performance *r* = 0.98 ± 0.02; mean ± SD; *N* = 17) and participants without a positive bias (mean bias = −1.2° ± 0.7°; performance *r* = 0.97 ± 0.03; *N* = 7). The difference in absolute perceptual bias between these groups was not significant (*t*_(22)_ = 1.74; *p* = 0.095); nor was the difference in performance (*t*_(22)_ = 0.82; *p* = 0.41). In participants with an expectation-induced bias in perception, the predictive cues evoked a significant motion direction signal from 135 to 175 ms poststimulus (peak = 26.3°; *p* = 0.044, *d* = 0.89), as well as from 270 to 340 ms (peak = 22.0°; *p* = 0.0054, *d* = 0.98; Fig. 4*B*). In the participants whose perception was not biased toward the predicted directions, there were no significant clusters in the MEG decoding signal (Fig. 4*C*). In fact, participants with a perceptual bias toward the predicted directions displayed a significantly stronger expectation signal from 245 to 355 ms than participants without such a perceptual bias (*p* = 0.0005, *d* = 1.93). Thus, individual variability in the neural signal evoked by the prediction cues was related to individual variability in the perceptual bias induced by these cues.

### Correlation between perceptual and neural representations emerges before stimulus onset

**Figure 5.**
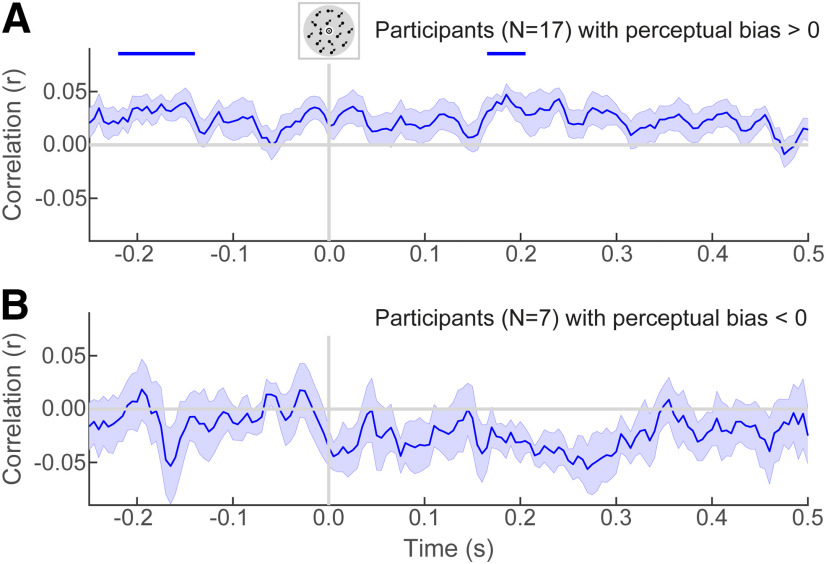
Single-trial correlation between reported and decoded directions. For each participant, the partial Pearson correlation across trials between decoded and reported directions, controlling for the presented direction, was calculated at each time step. ***A***, ***B***, The graph represents the mean of these within-participant correlation values for participants with a perceptual bias toward the cues (***A***) and for participants without a perceptual bias toward the cues (***B***). Shaded regions represent SEM. Cluster-based permutation tests established whether these correlation values were significant at the group level. Horizontal lines represent significant clusters resulting from these tests (*p* < 0.05).

To further elucidate the relationship between neural sensory representations and perception, we correlated the decoded direction from the MEG data with the perceived direction on individual trials, controlling for the presented direction (i.e., through partial correlation; see Materials and Methods). This analysis aimed to investigate whether fluctuations in neural representations were related to fluctuations in subjective perception. For the participants whose perception was biased toward the predictive cues (*N* = 17), decoded motion directions correlated significantly with perceived directions from −220 to 140 ms prestimulus (*p* = 0.0056, *d* = 1.07) and from 165 to 205 ms poststimulus (*p* = 0.023, *d* = 1.00; Fig. 5*A*). Thus, for these participants, the decoded neural signal was related to fluctuations in perception across trials. For the participants who did not display a perceptual bias toward the predictive cues (*N* = 7), there were no significant clusters (Fig. 5*B*). The correlation between decoded and perceived motion directions was significantly stronger for participants with a perceptual bias toward the predictive cues than for those without such a bias, from −10 to 35 ms (*p* = 0.041, *d* = 1.33) and from 160 to 315 ms (*p* = 0.0009, *d* = 1.94) poststimulus. In other words, the relationship between neural and perceptual fluctuations was stronger in participants for whom the predictive cues induced an attractive perceptual bias. In fact, these trial-by-trial correlations between decoded and perceived motion directions were not significant at the overall group level (i.e., without performing the participant split), suggesting that these effects were driven by the participants with a positive perceptual bias toward the directions predicted by the cues.

### Implicit nature of the expectations

Participants were not informed of the meaning of the auditory cues, and all participants filled out a debriefing questionnaire to assess whether they were aware of the predictive relationships. Seven of 24 participants indicated that they had noticed some relationship between the auditory tones and the direction of dots. Of these, only four correctly reported the true relationship, two reported the opposite relationship, and one was not able to report a specific relationship. The early neural effects of the predictive cues were still present when the four participants who reported being aware of the correct cue contingencies were excluded from the analysis (significant cluster from 125 to 190 ms poststimulus; *p* = 0.005, *d* = 0.92), indicating that the expectation effects did not depend on the subjects being aware of the predictive relationships.

### Eye movement control analysis

Eye movements are known to be able to confound MEG decoding analyses (Mostert et al., 2018). To minimize the effects of eye movements, we estimated our decoding model based on independent localizer runs during which participants were performing a central fixation task for which the directions of the moving dots were task irrelevant (Kok et al., 2017; Mostert et al., 2018).

Still, to investigate whether systematic eye movements during the localizer task could have affected our decoding results, we trained and tested the decoder on the vertical and horizontal gaze coordinates, as recorded by the eye tracker, rather than sensor amplitudes. Importantly, moving dot direction could not be decoded from the eye tracker signals in the first 150 ms poststimulus (*r* < 0.1 for all time points, no significant clusters at *p* < 0.05, also no single significant time point between 0 and 150 ms; *p* < 0.05 uncorrected). This is the critical time window for our analyses, since the decoder was trained on MEG data from the localizer runs ∼90–110 ms poststimulus. As an additional stringent control, we performed the main analysis of interest on the eye-tracking data; that is, training the decoding models on the eye tracker signals in the localizer data on individual peaks between 90 and 110 ms, and applying them to the eye tracker data from the main task to reveal any effects of the predictive cues. As expected, since participants did not make systematic eye movements during this time window in the localizer, this analysis yielded no significant cue effects (no clusters at *p* < 0.05; also no single significant time point between −250 and 500 ms; *p* < 0.05 uncorrected).

It is noteworthy that moving dot direction could be decoded from gaze position in the localizer at later time points, namely from 170 to 360 ms poststimulus (peak mean Pearson's *r* = 0.460, *p* < 0.001, *d* = 1.04). This indicates that participants moved their eyes systematically depending on the direction of the moving dots during this later time window in the localizer runs. Note, however, as discussed above, that there were no significant points in the earlier time window in which we trained the decoder for the MEG data (90–110 ms), meaning that these later eye movements did not affect our analyses.

As a final control analysis, we trained the decoding models on the eye tracker data from the localizer runs on individual peaks between 170 and 200 ms poststimulus (when localizer eye tracker decoding first became significant at the group level), and applied them to the eye tracker data from the main task. This analysis yielded no significant effects of the predictive cues on eye tracker signals (no clusters at *p* < 0.05; also no single significant time point between −250 and 500 ms; *p* < 0.05 uncorrected).

In sum, while the moving dot stimuli were shown to affect gaze position at a later time window during the localizer runs, we found no evidence that systematic eye movements could explain the effects of interest here.

## Discussion

There has recently been much debate as to whether expectations can alter early sensory processing (Summerfield and de Lange, 2014; De Lange et al., 2018) or instead only modulate later decision-making processes (Rao et al., 2012; Rungratsameetaweemana et al., 2018). Here, we find that implicit prior expectations can modulate sensory representations at an early stage, in line with suggestions that expectations play a fundamental role in sensory processing (Friston, 2005; Summerfield and de Lange, 2014). The latency of the effect (∼150 ms) suggests that this modulation may not affect the initial feedforward sweep of sensory processing, but rather occur once some recurrent processing has taken place in the visual system. However, importantly, the modulation is low level and sensory in nature, challenging proposals that expectations only modulate decision-making stages (Rao et al., 2012; Rungratsameetaweemana et al., 2018).

Perceptual reports of the direction of the moving dots were biased toward the direction predicted by the auditory cues. This is in line with previous studies (Chalk et al., 2010; Kok et al., 2013) as well as with theoretical work that casts perception as Bayesian inference, wherein the final percept is an integration of the prior expectations and perceptual input (Knill and Richards, 1996; Kersten et al., 2004). It is notable that expectations affected perception, although participants were not consciously aware of them (Chalk et al., 2010; Kok et al., 2013).

The effect of the predictive cues depended on the presented direction: expectations affected perception more strongly when the presented direction was oblique than when it was vertical or horizontal (Fig. 2). This may be because vertical and horizontal directions occur more frequently in natural environments, giving rise to “hyperpriors”: lifelong-learned expectations that these directions are likely to occur (Berkes et al., 2011; Girshick et al., 2011). Therefore, at directions closer to the cardinal directions, the experimentally induced priors may have interacted with hyperpriors, whereas at directions nearer 45° only the cue-related priors had an effect (Hu and Rahnev, 2019).

The primary motivation of this study was to establish whether expectations modulated the information content of early sensory signals. Predictive cues modulated the motion direction represented in the MEG signal as early as ∼150 ms poststimulus, as revealed by a decoder trained on early (∼100 ms) MEG signals evoked by task-irrelevant moving dots in separate runs.

The relatively early time point at which modulation occurs and the sensory nature of the signal are striking. The expectation signals were revealed by a decoder trained on task-irrelevant stimuli, isolating sensory processes common between the localizer and main task (Kok et al., 2014, 2017). This, together with the fact that the decoders were trained on early poststimulus time points, suggests that these expectation signals reflect sensory processing, rather than being related to later decisional, attentional, or motor processes (Mostert et al., 2015).

It should be noted that the latency at which expectation signals occurred (starting at 135 ms, peaking at 150 ms) is later than the first feedforward sweep of sensory information, which occurs within 50–80 ms (Clark et al., 1994; Alilović et al., 2019). The initial feedforward sweep of sensory information may therefore be model free, with expectations being integrated into the representation soon after (Marzecová et al., 2018; Alilović et al., 2019). Interestingly, many studies have found prestimulus effects of expectations (Kok et al., 2017; Samaha et al., 2018; Alilović et al., 2019) and attention (Myers et al., 2015), which do not seem to translate into subsequent modulations of the first feedforward sweep. The reason for this is not yet clear, but it has been suggested to be because of the fact that feedforward and feedback signals involve different neuronal populations (Bastos et al., 2012; Kok et al., 2016; Alilović et al., 2019).

Alternatively, the lack of an earlier modulation by expectations may have resulted from the type of stimulus used. Using motion rather than static stimuli means the accumulation of evidence takes inherently longer—indeed, within the localizer task, direction could not be decoded until 90 ms after stimulus onset, whereas studies using static stimuli have shown significant decoding in the localizer just 40–60 ms poststimulus (Cichy et al., 2014; Mostert et al., 2015; Kok et al., 2017). Furthermore, the decoding procedure used here aimed to distinguish several directions of motion within an ∼70° range (9° to 81°), rather than, for instance, decoding orthogonal orientations (Kok et al., 2017), which may have led to decreased signal-to-noise ratio, precluding successful decoding at earlier latencies.

In revealing expectation modulations of the information content of sensory signals at ∼150 ms poststimulus, our results accord with previous studies that report modulations of the amplitude of sensory signals by expectation at ∼100–150 ms (Bar et al., 2006; Meyer and Olson, 2011; Todorovic et al., 2011; Wacongne et al., 2011; Todorovic and de Lange, 2012; Stojanoski and Niemeier, 2015; Aru et al., 2016; Samaha et al., 2018) or even earlier (Keil et al., 2017). However, they conflict with experiments reporting no effects of expectation on early sensory processing (Rao et al., 2012; Rungratsameetaweemana et al., 2018). An important difference between studies may be the extent to which subjects form a perceptual expectation. For instance, in a recent study in macaques (Rao et al., 2012), the expectation cue predicted both which stimulus would appear, as well as the correct response. Since accurate task performance was strongly incentivized, the cue may have induced a response bias, rather than a perceptual bias. In the present study, to avoid strategic guessing or response bias, participants were not informed of the predictive relationship between the cues and the motion direction. In a recent study in humans using EEG that failed to find the effects of expectation on sensory processing (Rungratsameetaweemana et al., 2018), the authors likely also predominantly manipulated task expectations, rather than perceptual expectations. That is, expectations pertained not so much to the statistics of the upcoming sensory inputs per se, but more so to which features of the inputs were likely to constitute a target. Therefore, this study more strongly manipulated task set expectations than perceptual expectations.

A noteworthy aspect of our central finding is that the neural motion direction signal induced by the predictive cues peaked at ∼20°, rising to 26° when considering only participants with a positive perceptual bias. This difference is an order of magnitude greater than the mean perceptual bias, being instead closer to the angle difference between the directions predicted by the two cues (63° vs 27°). This suggests that this early neural effect may reflect a reactivation of the predicted direction, rather than the integration of the predicted and presented directions. This is in line with the fact that this neural expectation effect peaks at ∼150 ms and then reduces, perhaps reflecting the integration of an expectation template (Kok et al., 2014, 2017) with incoming sensory evidence.

Intriguingly, a *post hoc* split of the participants based on their perceptual bias indicated that, for the participants who showed a perceptual bias toward the predictive cues (*N* = 17), the neural expectation effect reappeared at ∼300 ms (Fig. 4*B*). One possibility is that this reflects periodic activation of top-down expectations during recurrent feedforward and feedback message passing. Theoretical work that characterizes perception as predictive processing postulates cycles of processing alternating between feedforward and feedback information, with the current hypothesis being tested and then iteratively revised until the hypothesis matches the incoming sensory information (Knill and Pouget, 2004; Friston, 2005; Bastos et al., 2012). Such recurrent message passing has recently been shown to occur at a frequency of ∼11 Hz during perception (Dijkstra et al., 2019).

Decoding analyses can reveal whether certain information is present in the neural signal, but not whether this information is part of the perceptual process or is merely epiphenomenal. One way to address this inferential gap is to verify whether the decoded signal is related to behavioral variation (de-Wit et al., 2016). We found such a relationship both across and within participants. Across participants, neural expectation signals were stronger in participants with an expectation-induced perceptual bias. For participants with such a perceptual bias, decoded and perceived directions were correlated across trials even before stimulus onset. This finding is in line with previous work suggesting the prestimulus state of the sensory cortex biases perception (Hesselmann et al., 2008; Pajani et al., 2015; Sherman et al., 2016; Han and VanRullen, 2017; Kok et al., 2017; Gandolfo and Downing, 2019). In short, the neural signal explained behavioral variation over and above that explained by the physical stimulus (Kok et al., 2013; St John-Saaltink et al., 2016). Given that both of these neural–behavioral relationships were demonstrated in exploratory analyses in which participants were split *post hoc*, future studies will need to replicate these findings to ensure their robustness.

In summary, our results demonstrate that expectations modulate the information content of sensory signals early on in the perceptual process. These findings concord with predictive processing theories of perception that posit that expectations are a fundamental constituent of early sensory processing (Lee and Mumford, 2003; Friston, 2005; Summerfield and de Lange, 2014; Keller and Mrsic-Flogel, 2018).
